# ﻿Relationship of Trichoptera species in Iceland with Europe and North America

**DOI:** 10.3897/zookeys.1263.148150

**Published:** 2025-12-10

**Authors:** Gísli Már Gíslason, Snaebjörn Pálsson

**Affiliations:** 1 Institute Life and Environmental Sciences, University of Iceland, Askja - Natural Science Building, Sturlugata 7, IS-102 Reykjavik, Iceland University of Iceland Reykjavik Iceland

**Keywords:** COI mDNA, genetic relationship, linages, phylogeography, post-glacial colonisation

## Abstract

In the present study we examine the geographic variation in the COI mtDNA barcode marker in eight Trichoptera species from Iceland to determine their postglacial colonisation history. The patterns in 10 of 12 Trichoptera species found in Iceland indicate distinct histories where different species show varying time since colonisation of the island and separate evolution restricted to Iceland. The Holarctic *Limnephilus fenestratus* and *L. picturatus* do not show a clear split between the Nearctic and Palaearctic. As previously reported for the parthenogenic *Apatania zonella* in Iceland, the species was found to have colonised the island both from the Nearctic and Palaearctic. Four of the Palaearctic species *L. affinis*, *L. griseus*, *L. sparsus*, and *L. elegans* present unique lineages in Iceland, suggesting that they were early colonisers after the last glacial period of Ice Age or during Holocene. Variation within the three other Palaearctic species reflect a recent origin. *Limnephilus decipiens* is a late coloniser, first recorded in 1929 in one location, and *Micropterna sequax*, a very recent coloniser in the 21^st^ century, share identical genetic sequences with other European specimens, as previously reported for *Potamophylax cingulatus*, which is known to have colonised Iceland in the last century. Five of the 10 species suggest unique mtDNA lineages in Iceland and early colonisation.

## ﻿Introduction

During the last glacial maximum of the Ice Age 25 Kyr ago, all of Iceland was covered with ice and this ice cover extended up to 100 nautical miles beyond the present coastline (Geirsdóttir et al. 2009, 2013). The last glacial period of the Ice Age abruptly ended 11 Kyr ago, when the temperature rose in the North Atlantic islands within a few decades from severe coldness to a climate warmer than today (Dansgaard et al. 1993; Geirsdóttir et al. 2013). No terrestrial or freshwater life are known to have survived this condition in Iceland, except for two subterraneous groundwater amphipod species (Kristjánsson and Svavarsson 2007; Kornobis et al. 2010, 2011). The present Trichoptera fauna is, therefore, post-glacial.

The Icelandic fauna is mostly of Palaearctic origin, with only a few species of Holarctic or Nearctic origin (Downs 1988; Gíslason 2005, 2021). The Greenland glacier appears to be a great barrier to dispersal for American insects and other animals migrating east to Iceland. During the glacial periods of the Pleistocene, many terrestrial species at high latitudes in Europe regressed to refugia at southern latitudes where over a long time they diverged in allopatry from one other (Hewitt 2000, 2004). Such putative refugia have been described in the Iberian Peninsula, Italy, and the Balkans (Taberlet et al. 1998; Hewitt 2000). The genetic variation of many species reflects these climatic changes of the Pleistocene on the population range and size, where little variation is commonly found within regions but with sharp boundaries between regions (Hewitt 2000, 2004).

Iceland is known for its low diversity of insects (Gíslason 2005), as insects must rely on their own flight strength for transport across the Atlantic or an infrequent passive dispersal. Thus, aquatic insects in Iceland have much lower species diversity than Cladocera, for example, which are transported with waterfowl. About 30% of the cladoceran fauna of Britain and Norway is found in Iceland, but only 6% of the aquatic insects of these regions occur in the country (Gíslason 2005, 2021). Only 12 caddisfly species have been recorded from Iceland (Gíslason 1981b; Ólafsson and Gíslason 2010), compared with about 200 in the nearest regions of Norway and Britain. The colonisation of Iceland by caddisflies and other insects is still in progress (Gíslason 1974; Ólafsson and Gíslason 2010). In our recent studies on phylogeographic variation on two caddisflies, *Apatania zonella* (Pálsson et al. 2016) and *Potamophylax cingulatus* (Gíslason et al. 2015) two distinct patterns were found. In *A. zonella* high variation was found in mtDNA in Iceland, which clustered into two separate lineages, which have diverged separately 240–450 Kyr ago, one in the Palaearctic and one in the Nearctic, which suggests colonisations from each of these regions. A unique clade in the Palaearctic branch is observed in Iceland, which shared a common ancestry with other sequences around 50 Kyr ago or during the last glacial period of Ice Age. The unique lineage in Iceland may have diverged elsewhere before colonisation of Iceland and may have either disappeared elsewhere or not been described because of limited geographic sampling. The second lineage in Iceland of *A. zonella* shared identical sequences with specimens from southern Greenland where Icelanders had settled in 985 AD, suggesting a geneflow during that time, or later, between the two islands. The phylogeography observed for *P. cingulatus*, a relatively recent coloniser in Iceland (Gíslason 1974; Gíslason et al. 2015), is quite different (Gíslason et al. 2015). Within Europe the mtDNA COI variation confines three monophyletic lineages which matches three subspecies, where the Icelandic lineage has identical COI sequences as found in the Faroe Islands, in agreement with the recent colonisation and sharing close similarity to samples in Western Europe, which have diverged from Spanish samples. The dispersal rate of *P. cingulatus* was estimated around 7–9 km/year, and at a site where the species had become established, there was still exponential growth of the population 26 years after its first record (Gíslason et al. 2023).

Research on the origin of other freshwater invertebrates in Iceland is scarce. Aside the aforementioned studies on two species of caddisflies (Gíslason et al. 2015; Pálsson et al. 2016) and on groundwater amphipods (Kornobis et al. 2010, 2010), the phylogeography of *Daphnia pulex* (Weider et al. 1996) and *Radix balthica* (Bolotov et al. 2017) show different patterns, where the latter has a unique mtDNA lineage in Iceland. Research on plant dispersal in the Arctic show that the initial colonisation after the Ice Age was by drift ice (Alsos et al. 2016), and Beringia served as both a refugium and a source for interglacial colonisation and recolonisation, whereas areas further west in Siberia served as refugia, but less as sources for colonisation and recolonisation (Eidesen et al. 2013).

In the present study we will examine the geographic variation in the COI mtDNA barcode marker in eight Trichoptera species from Iceland and compare with the patterns observed for *Apatania zonella* and *Potamophylax cingulatus* described above. Unique clades or lineages within the species in Iceland will support longer time since colonisation of Iceland as found for *A. zonella*, whereas close similarity to specimens from other areas recent origin as in *P. cingulatus*. Two of the species studied, *Limnephilus fenestratus* and *L. picturatus*, have a circumpolar distribution similar to *A. zonella*, whereas six species, *L. affinis*, *L. elegans*, *L. griseus*, *L. sparsus*, *L. decipiens*, and *Micropterna sequax*, have a Palaearctic or European distribution similar to *P. cingulatus*. *M. sequax* recently colonised Iceland and has only been found at two locations in southwest Iceland. Five species, *L. affinis*, *L. fenestratus*, *L. griseus*, *L. picturatus*, and *L. sparsus* are distributed all over Iceland (Gíslason 1981b), and *P. cingulatus* has now almost reached all regions of Iceland (Gíslason et al. 2023). Of the other species in this study, *L. decipiens* and *L. elegans* have a distribution limited to areas where the average July temperature is over 10 °C (Gíslason 1981b), and *L. decipiens* has recently extended its distribution from southern to northern Iceland (Gíslason pers. com.).

Information on the COI mtDNA haplotypes barcode regions of the two other species of Trichoptera recorded in Iceland were not available. They are *Grammotaulius nigropunctatus* (Limnephilidae) and *Agrypnia picta* (Phryganeidae), both with a Palaearctic distribution.

The results presented here indicates distinct histories for each of the species and that their colonisation of the island is still ongoing.

## ﻿Materials and methods

Adults and larvae of the eight Trichoptera species were collected between 2005 and 2012 from various locations in Iceland, Greenland, and the Faroe Islands. We collected adults during the flight period (early May–late August) with nets and traps and larvae from stones in streams and rivers.

We preserved the samples individually in 96% ethanol. Species were identified based on morphological characteristics under a stereomicroscope. Specimens were sent to Karl Kjer at the iBOL consortium (https://ibol.org/) for DNA barcoding, where DNA extraction, PCR amplifications using primers LCO1490 and HCO2198 (Folmer et al 1994), and sequencing were conducted. Additionally, COI-barcode sequences from Iceland and other countries were downloaded in May 2023 from the BOLD database (https://v3.boldsystems.org/) (Ratnasingham and Hebert 2007). Accession numbers, sequence length, and country of origin are shown in Suppl. material 1. Most sequences were of 658 bp, but shorter sequences (>600 bp) were also included.

### ﻿Molecular analysis

Where sequences from a particular species were more similar to those of another species, there sequences were omitted; these cases appear to have resulted from either wrong identifications or either ancient polymorphism or hybridisation.

### ﻿Phylogeographical analysis

Maximum-likelihood trees were reconstructed using Phyml implemented in SeaView (Gouy et al. 2010) and redrawn using the APE package (Paradis 2004) in R (R Core Team 2023). *Limnephilus griseus* was used as an outgroup, except for its phylogeny when *L. sparsus* was used. The effect of having two outgroup species was evaluated and when a difference in the topology was observed both species were used, as listed. The outgroups were removed from the trees presented for the visualisation, but the distance to the outgroups is given in the figure legends. In SeaView the maximum-likelihood estimation of trees was estimated with different evolutionary models of nucleotide substitutions and the model selected which gave the tree with the highest likelihood. Empirical frequencies of the nucleotides were used and rate-variation across sites was optimised for four rate categories. Nearest-neighbour interchange (NNI) was used for tree searching operation, starting with a neighbour-joining tree (BioNJ), optimised for topology and five random starts. The support for branches within the phylogenetic tree was evaluated with the approximate likelihood ratio test (aLRT) (Anisimova et al. 2006). Cophenetic distances between the outgroup and the sequences within species were calculated with APE. A strict clock of 2% divergence per million years was used to estimate the divergence time for the COI barcode region, focusing especially on the divergence of Icelandic sequences from the others.

## ﻿Results

**Figure 1.**
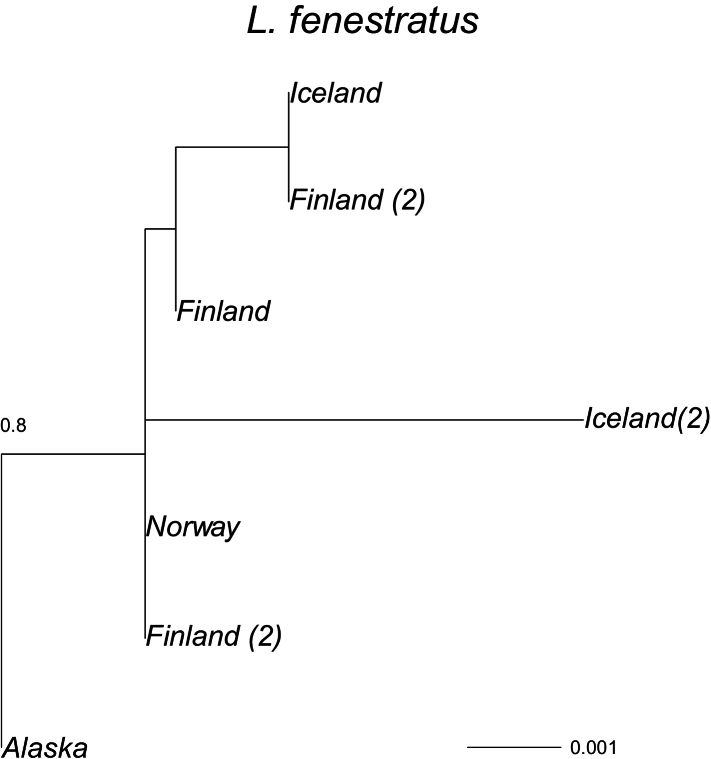
A maximum-likelihood phylogeny based on the COI mtDNA haplotypes (barcode region) for *Limnephilus fenestratus*. Tip labels refer to geographical origin with frequencies (>1) of unique haplotypes in regions given in brackets. Scale bar presents a divergence in proportion of base pair changes. *Limnephilus griseus* and *L. affinis* were used as outgroups. Its average distance from the sequences presented were respectively 0.180 and 0.194.

**Figure 2.**
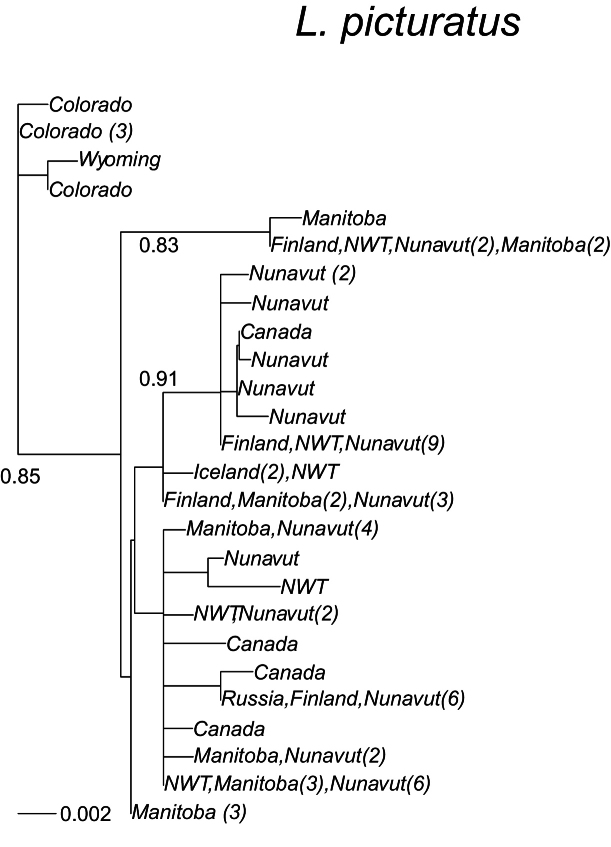
A maximum-likelihood phylogeny based on the COI mtDNA haplotypes (barcode region) for *Limnephilus picturatus*. Tip labels refer to geographical origin with frequencies (>1) of unique haplotypes in regions given in brackets. Scale bar presents a divergence in proportion of base pair changes. *Limnephilus coloradensis* and *L. griseus* was used as an outgroup. Their average distances from the sequences presented were 0.129 and 0.099, respectively.

The phylogenetic tree (Fig. 1) for *Limnephilus fenestratus* based on GTR distances, where all substitutions rates are estimated, gave the highest likelihood (-log likelihood GTR = 1140.9, F84 = 1151.9). The phylogenetic tree (Fig. 2) for *L. picturatus* based on GTR distances, where all substitutions rates are estimated, gave the highest likelihood (-log likelihood GTR = 1660.9, F84 = 1674.7). The circumpolar *L. fenestratus* (Fig. 1) and *L. picturatus* (Fig. 2) present different phylogenies, but these suggest that there may have been recent migration of individuals to Iceland, as Icelandic sequences of these species are identical as in other countries.

*Limnephilus fenestratus* has been poorly sampled geographically and only 10 sequences are found in the BOLD database. A split is observed between the single sample from Alaska and the European samples with an average distance of 0.003. Two haplotypes are observed in Iceland (Fig. 1): one is identical to a sequence obtained from a specimen from Finland (*d* = 0), which clusters with specimens from Norway and Alaska, whereas two specimens from Iceland differ from the other European sequences by a distance of 0.005, which may suggest an older settlement of the species in Iceland.

Many *Limnephilus picturatus* COI sequences exist in the BOLD database but only few of them are from Europe. One *L. picturatus* sequence (GenBank no. KX293667.1) was identical to *L. coloradensis* (GenBank no. GU667953.1) and another with BOLD number HM395682 was highly similar to *L. griseus*. Average distances within *L. picturatus* was 0.003.

Two specimens from Iceland have identical sequences to ones from Northwest Territories in Canada, and specimens from Finland have also identical sequences as found in Canada (Fig. 2). A unique clade is confined by specimens in Colorado and Wyoming, but otherwise no sign of geographical structure is observed in the phylogeny. Most of the samples originate from North America, and more samples are needed from Europe.

**Figure 3.**
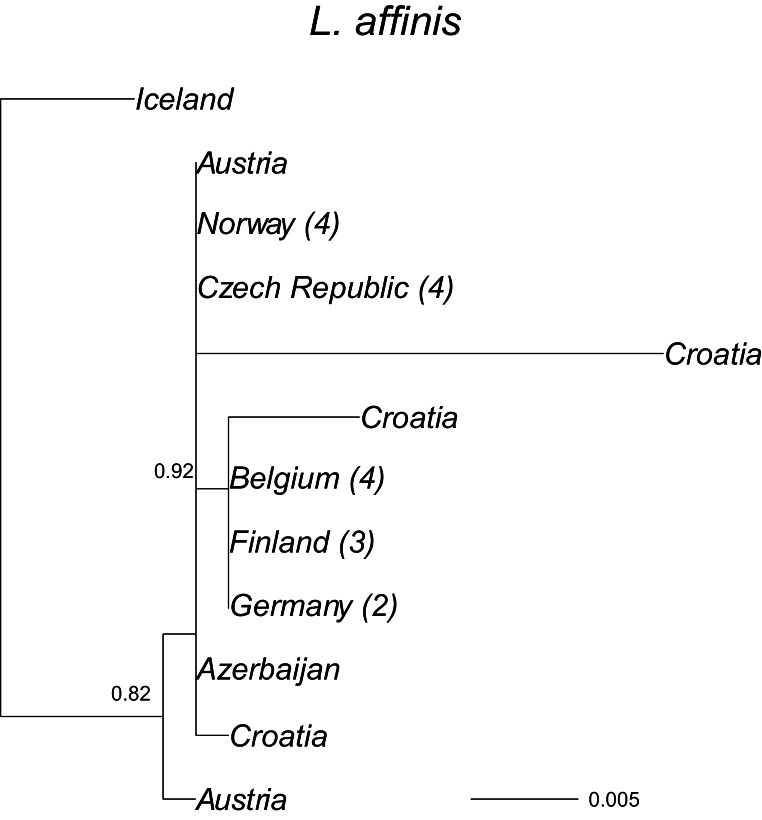
A maximum-likelihood phylogeny based on the COI mtDNA haplotypes (barcode region) for *Limnephilus affinis*. Tip labels refer to geographical origin with frequencies (>1) of unique haplotypes in regions given in brackets. Scale bar presents sequence divergence in proportion of base pair changes. *Limnephilus griseus* was used as an outgroup; its average patristic distance from the other sequences was 0.135.

The phylogenetic tree for *Limnephilus. affinis* based on GTR distances, where all substitutions rates are estimated gave the highest likelihood (-log likelihood GTR = 1546.5, F84 = 1558.9) (Fig. 3). The average patristic distance of the Icelandic *L. affinis* from the other samples was 0.017, with a proportion of sites of different sites between sequences of 0.014. This suggests an independent evolution of the mtDNA sequences in Iceland or even before colonisation, whereas the average distance among the other European samples was 0.002.

In the small sample of *Limnephilus elegans* a similar pattern is observed but the distances are smaller. A maximum likelihood tree was obtained with the GTR model (-log likelihood = −1172.5, F84 = −1199.0). The distance of the Icelandic specimens from the others within the species is 0.0015, with the same raw distance, whereas the other sequences are identical.

The likelihood of the tree for *Limnephilus griseus* was highest based on GTR distances (-log likelihood = 1209.6, with F84 = −1216.5). *L. griseus* (Fig. 5) seems to have had two colonisation events in Iceland, with average distances of 0.002–0.005 from the others and one more different (and initially classified as *L. picturatus* based on morphology) with a distance of 0.009–0.012 (range of raw distances 0.002–0.011), whereas distances among other samples from Europe ranged from 0.000 to 0.008 (mean = 0.001).

**Figure 4.**
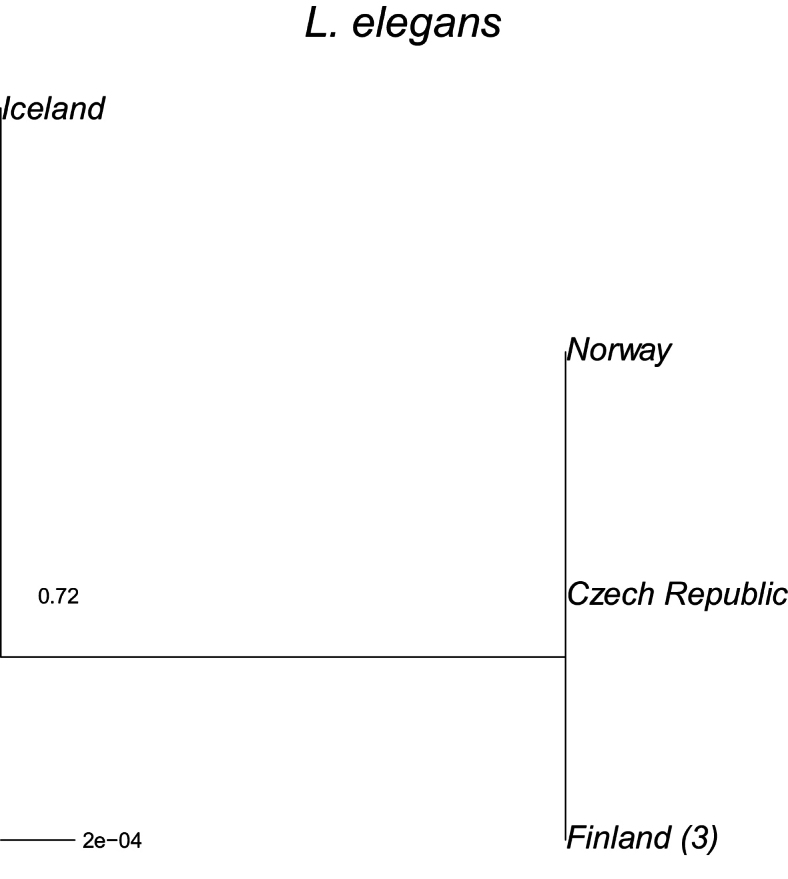
A maximum-likelihood phylogeny based on the COI mtDNA haplotypes (barcode region) for *Limnephilus elegans*. Tip labels refer to geographical origin with frequencies (>1) of unique haplotypes in regions given in brackets. *Limnephilus griseus* was used as an outgroup. Its average patristic distance from the sequences presented was 0.152.

**Figure 5.**
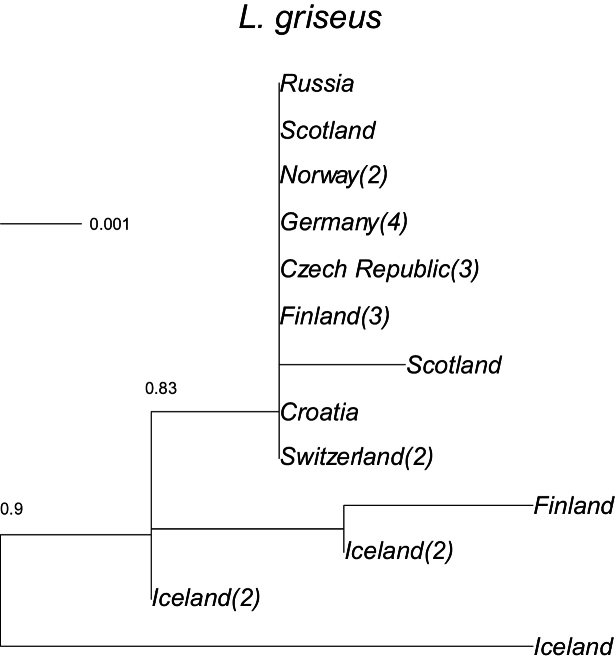
A maximum-likelihood phylogeny based on the COI mtDNA haplotypes (barcode region) for *Limnephilus griseus*. Tip labels refer to geographical origin with frequencies (>1) of unique haplotypes in regions given in brackets. Scale bar presents sequence divergence in proportion of base pair changes. *Limnephilus sparsus* was used as an outgroup, its average distance from the sequences presented was 0.124.

**Figure 6.**
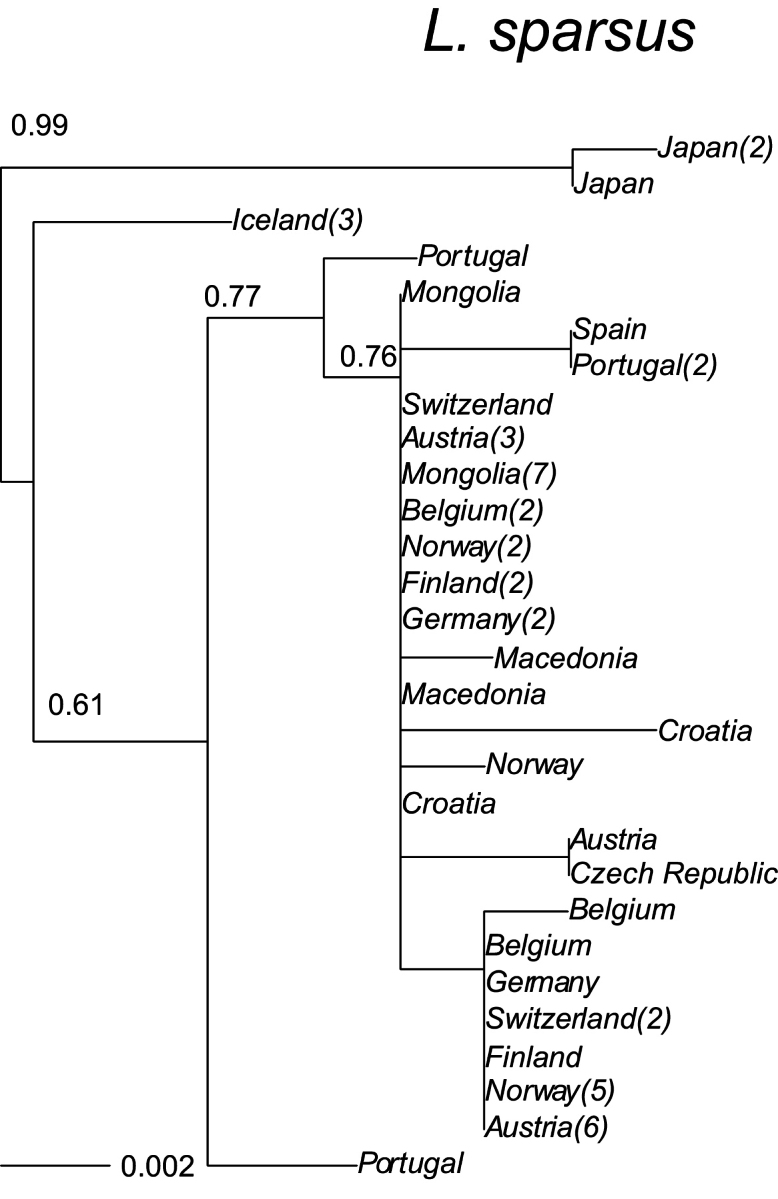
A maximum-likelihood phylogeny based on the COI mtDNA haplotypes (barcode region) for *Limnephilus sparsus*. Tip labels refer to geographical origin with frequencies (>1) of unique haplotypes in regions given in brackets. Scale bar presents a divergence in proportion of base pair changes. *Limnephilus griseus* and *L. affinis* were used as outgroups. The average of the outgroups from the sequences presented were 0.183 and 0.166 respectively.

The likelihood of the tree (Fig. 6) for *Limnephilus sparsus* was highest based on GTR distances (-log likelihood = −1503.8, with F84 = −1513.9). Distances from Icelandic *L. sparsus* from the other samples in Europe were 0.009–0.014 (mean 0.010). Distances among countries within Europe ranged from 0.000 to 0.011 (mean = 0.002). The samples from the Scandinavian populations show close affinities to other samples within the same species from mainland Europe, and the distances were smaller than for the Icelandic species.

**Figure 7.**
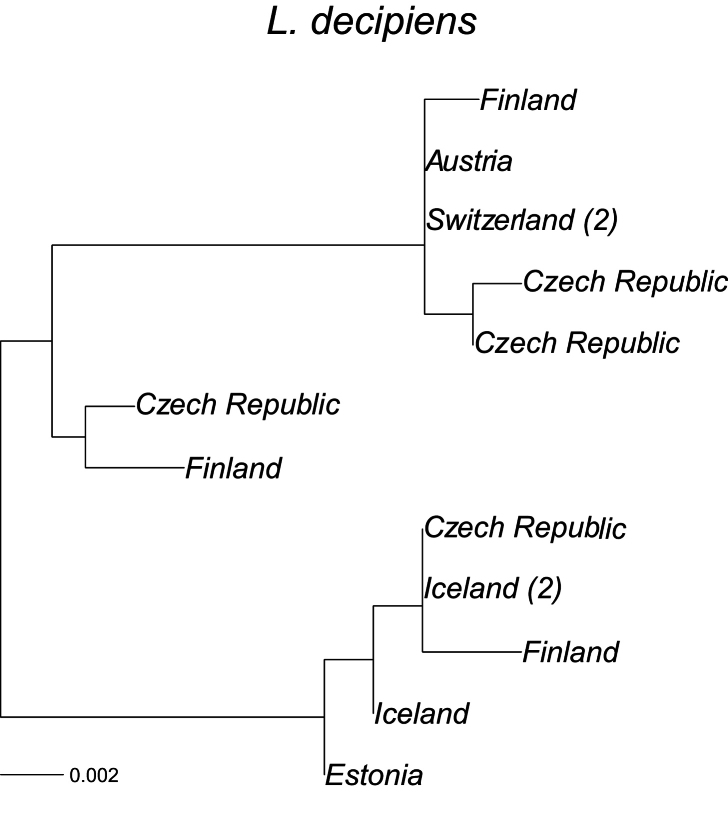
A maximum-likelihood phylogeny based on the COI mtDNA haplotypes (barcode region) for *Limnephilus decipiens*. Tip labels refer to geographical origin with frequencies (>1) of unique haplotypes in regions given in brackets. Scale bar presents a divergence in proportion of base pair changes. *Limnephilus griseus* was used as an outgroup. Its average distance from the sequences presented was 0.202.

The likelihood of the tree (Fig. 7) for *Limnephilus decipiens* was highest based on GTR distances (-log likelihood = −1336.7, with F84 = −1343.5, but the topologies of the trees were identical. Two distinct mtDNA clades are found within *L. decipiens*; they are well separated by a distance of 0.022, which could represent cryptic species within Europe, with distances within clades being 0.002 and 0.008. The Icelandic samples cluster within the clades with lower variation. Two sequences from Iceland are identical to a sequence from the Czech Republic (Fig. 7).

**Figure 8.**
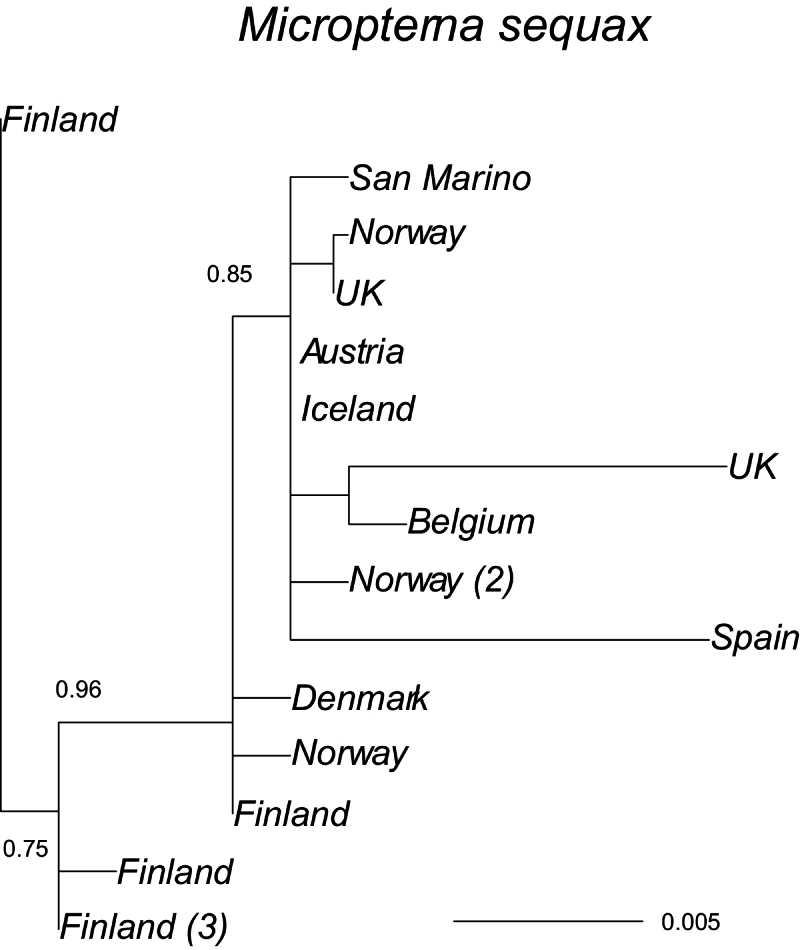
A maximum-likelihood phylogeny based on the COI mtDNA haplotypes (barcode region) for *Micropterna sequax*, rooted with *L. griseus*. Tip labels refer to geographical origin with frequencies (>1) of unique haplotypes in regions given in brackets. Scale bar presents a divergence in proportion of base pair changes. *Limnephilus griseus* was used as an outgroup. Its average distance from the sequences presented was 0.192.

The likelihood of the tree (Fig. 8) for *Micropterna sequax* was highest based on GTR distances (-log likelihood = −1379.4, with F84 = −1387.7), but the topologies of the trees were identical. *M. sequax* does not have unique lineage in Iceland and Icelandic material has sequences identical to those from Austria; this suggests a recent colonisation of Iceland by this species. There is no clear structure apparent within Europe, although the sample size is small, except for the similarity among the Scandinavian samples and the interestingly large divergence within Finland. The average distance within Europe is 0.007.

## ﻿Discussion

Iceland is still in the process of post-glacial colonisation by insects. By 1931, 10 species of Trichoptera were known from Iceland (Lindroth 1931). Two species have since been recorded, *Potamophylax cingulatus* (Gíslason 1974), with the oldest specimen found in Iceland in 1959, and *Micropterna sequax* (Ólafsson and Gíslason 2010, 2023), with the first specimens found in 2008. It is unlikely that large species of Trichoptera, like *P. cingulatus* or *M. sequax*, could have escaped the notice of entomologists, who have been able to find small caddisfly species, such as *Apatania zonella. A. zonella* has been replaced by *P. cingulatus* in the streams in the north-eastern and eastern Iceland in 1974–1978 by predation (Gíslason 1981a), and it is now restricted to streams and rivers in the west and south (Pálsson et al. 2016), although it might be present on lake shores in other areas of Iceland. Analysis of geographic variation in the COI mtDNA barcode marker in Trichoptera species from Iceland indicates distinct histories where time since colonisation of the island vary and evolution has been restricted to Iceland and separate from other areas.

Icelandic samples of *Potamophylax cingulatus* and *Micropterna sequax*, two species, recently originating from the Palaearctic, show no differentiation of mainland Europe samples (Gíslason et al. 2015; present study). The COI mtDNA gene in *P. cingulatus* could have diverged in southern refugia during the last glacial period east and southeast of the Alps and in Spain. Following the retreat of the glaciers the descendants of Spanish population presumably expanded north to Britain and Norway, and onwards to the west to the Faroe Islands and finally to Iceland, which was presumably colonised in the mid-20^th^ century (Gíslason et al. 2015). *M. sequax* was first recorded as *M. lateralis* (Ólafsson and Gíslason 2010) but later corrected (Ólafsson and Gíslason 2023). The COI sequence in *M. sequax* from Iceland did not differ from a specimen sampled in Austria, which lends support that the Icelandic population is the result of a very recent colonisation. There is also no clear geographic structure within Europe based on the marker. Icelandic *Limnephilus decipiens*, which shares identical Czech sequences, is also a relatively recent newcomer, although it has been known in Iceland for nearly a century (Lindroth 1931). The first four specimens of *L. decipiens* were found in 1929 (Lindroth 1931) in a single location in southern Iceland and by 1937 seven specimens had been recorded at two locations in southern Iceland (Fristrup 1942). In 1974–1975 this species’ distribution was restricted to the lowlands of southern Iceland and 36 additional specimens had been recorded (Gíslason 1981b). The present-day distribution is the lowlands in southern, northern, and eastern Iceland, with a further 350 specimens recorded (Natural Science Institute of Iceland Database). The other species, *L. affinis*, *L. elegans*, *L. griseus*, and *L. sparsus* (Figs 3–6), all have unique lineages in Iceland, indicating colonisation during different periods of the Holocene; the species may have colonised Iceland shortly after the end of the last glacial period. Considering their genetic distances, *L. affinis* and *L. sparsus* may have been in Iceland for the longest time, or they have been colonised by mtDNA lineages not yet observed elsewhere. *Limnephilus griseus* seems to have twice colonised Iceland: an early first colonisation and again more recently.

The circumpolar *Limnephilus fenestratus* and *L. picturatus* (Figs 1, 2) do not show any evidence of a long presence in Iceland. Sequences identical to the ones found in Iceland were also found in Finland (*L. fenestratus*) and the Northwest Territories of Canada (*L. picturatus*). Two *L. fenestratus* sequences from Iceland differed the most from the other samples, possibly indicating an earlier colonisation. However, the distance was not large. Also, the sample size from Iceland is very small for both species, with three and two specimens, respectively. Thus, an interpretation is difficult. This pattern differs from the pattern observed in the circumpolar almost-asexual *Apatania zonella*, which a showed considerable variation in the COI mtDNA barcode marker and shared variation with other species of *Apatania* (Pálsson et al. 2016). Pálsson et al. (2016) found two distinct linages in Iceland, one associated with Greenland, and another with Scandinavia. Also, appears that the lineage originating in the Palaearctic has been present for a long time in Iceland, whereas the Nearctic lineage is a recent coloniser. As hypothesised in Pálsson et al .(2016), it could have been brought by humans from Greenland during 1000–1400 AD.

The close similarity of the mtDNA sequences within four species and no distinct lineage from European populations suggest that the post-glacial colonisation is still ongoing, which is supported by the observed and tracked colonisation of two of 12 species in Iceland during the last 70 years. The analysis presented here is based on only a single mtDNA marker and in some cases a geographically limited dataset. Yet, the data still reveals interesting patterns where distances to Icelandic specimens are often the largest distances within the species or in Europe, suggesting that the geographic isolation of Iceland has led to divergence and maintenance of separate lineage in Iceland which contribute to the diversity within these species.

## ﻿Conclusion

Post-colonisation in Iceland is still in progress. Some species appears to have been on the island for a long time, possibly since shortly after the end of the last Ice Age (*Apatania zonella*, *Limnephilus affinis*, *L. griseus*, and *L. sparsus*), whereas *L. decipiens* appears to be a relative newcomer. *Limnephilus fenestratus* and *L. picturatus* (Figs 1, 2) do not show any evidence of long existence in Iceland, and identical sequences were found in Iceland as in Finland (*L. fenestratus*) and the Northwest Territories, Canada (*L. picturatus*). Two species arrived during the last decades (*Micropterna sequax* and *Potamophylax cingulatus*). At least two species seem to have colonised Iceland twice: *A. zonella* recently (around 1000 years ago) from North America and early after last glacial period from Europe, and *L. griseus* twice from Europe.

## ﻿Additional information

### Author ORCIDs

Gísli Már Gíslason https://orcid.org/0000-0001-8507-1796

Snaebjörn Pálsson https://orcid.org/0000-0002-4297-3500

### Data availability

Genome sequence data are available from life database (BOLD). GenBank and BOLD numbers are given in Supplementary Information.

## Supplementary materials

Supplementary material 1 Relationship of Trichoptera species in Iceland with Europe and North America Data type docx Explanation note Cytochrome oxidase I sequences retreived from Genbank and the Bold database from eight Trichopteran species and their respective accession numbers. https://binary.pensoft.net/file/1488237This dataset is made available under the Open Database License (http://opendatacommons.org/licenses/odbl/1.0/). The Open Database License (ODbL) is a license agreement intended to allow users to freely share, modify, and use this Dataset while maintaining this same freedom for others, provided that the original source and author(s) are credited.Gísli Már Gíslason,Snaebjörn Pálsson
