# Supplementary material for: ﻿Relationship of Trichoptera species in Iceland with Europe and North America
Source: Zookeys. 2025 Dec 10;1263:89–104. doi: 10.3897/zookeys.1263.148150 (PMC12712638; doi:10.3897/zookeys.1263.148150)
Supplement: Supplementary material 1 — Relationship of Trichoptera species in Iceland with Europe and North America [file zookeys-1263-089_article-148150__-s001.docx]

**Supplementary table**

**Relationship of Trichoptera species in Iceland with Europe and North America**

Gísli Már Gíslason and Snaebjörn Pálsson

**Table S1.** Cytochrome oxidase I sequences retrieved from Genbank and the Bold database from eight Trichopteran species and their respective accession numbers. NA: not available.

| **Species** | **Country** | **GenBank accession no.** | **Bold accession no.** |
| --- | --- | --- | --- |

| *Limnephilus fenestratus* | Alaska | JQ907884 | OFCAD591N08 |
| --- | --- | --- | --- |
|  | Finland | HM902369, KX292692 KX296115, KX296220 NA | FICAD042N10, FICAD043N10, TRIFI1161N15, TRIFI1162N15, TRIFI1163N15 |
|  | Iceland | HM395680, HM395683, NA | ICEFI004N17, KKCAD567N09 ,KKCAD571N09 |
|  | Norway | NA | ZMBN641N17 |
| *Limnephilus picturatus* | Colorado, USA | NA | DRCAD023-08, DRCAD027-08, GBMIN91734-17, OFCAD715-08, OFCAD716-08 |
|  | Finland | NA | TRIFI1152-15, TRIFI361-11, TRIFI687-12, TRIFI899-13 |
|  | Iceland | NA | ICEFI005-17, KKCAD569-09 |
|  | Manitoba, Canada | NA | CUTLV367-09, CUTLV368-09, DSTRI579-07, DSTRI580-07, DSTRI581-07, DSTRI582-07, EBTCH145-11, EBTCH338-11, EBTCH340-11, EBTCH342-11, EBTCH345-11, MHTRI006-06, MHTRI010-06 |
|  | Northwest Territories, Canada | NA | NTUA3702-14  CNTUA3704-14, CNTUA5543-14, CNTUA5548-14, CNTUA5550-14, CNTUB5652-1 |
|  | Nunavut, Canada | NA | ACHAR1285-18, ACHAR2052-18, ACHAR2053-18, ACHAR3217-19, ARCAQ087-08, ARCAQ115-08, ARCAQ222-08, ARCAQ394-08, BCHAR1969-18, BCHAR1972-18,BCHAR2664-18, DCHAR1016-19, DNARA3649-21, DNARA3660-21, DNARA3664-21, DNARA3665-21, DNARA3666-21, DNARA3671-21, DNARC497-19, DNARC509-19, FCHAR2154-19, FCHAR2759-19, FCHAR5180-19, FCHAR6948-19, FCHAR6949-19, FCHAR6950-19, FCHAR9375-20, GCHAR349-19, GCHAR802-19, GCHAR846-19, KUGA229-21, KUGA231-21, KUGA233-21 KUGA234-21  KUGA235-21 MCHAD1675-19  POROM011-19, POROM133-19, POROM134-19 |
|  | Canada, unspecified | NA | GBMH17930-19, GBMH17988-19, GBMH18004-19, GBMH18232-19, GBMH18269-19, GBMH18271-19 |
|  | Russia | NA | GBMNB60796-20 |
|  | Wyoming, USA | NA | KJTRI548-13 |
| *Limnephilus affinis* | Austria | KX292193, KX142995 | BHMKK211-12, HMKKT848-11 |
|  | Azerbaijan | HM395028, NA | IRCAD007-10, TRIJS034-18 |
|  | Belgium | KX140824, KX142144 ,KX143567, KX144786 | TFLAN165-11, TFLAN166-11, TFLAN187-11 ,TFLAN275-11 |
|  | Croatia | NA | CROAA042-18, CROTR032-19 |
|  | Czech Republic | HQ958962, HQ958975, HQ959161, HQ959162, KX291336 | BHMKK173N12 ,HMKKT064-10, HMKKT078-10, HMKKT359-10, HMKKT360-10 |
|  | England | NA | UKAN4711-24 |
|  | Finland | HM902372, KX141248, KX293431, KX294590, KX296597 | FICAD046-10, TRIFI1129-15, TRIFI1130-15, TRIFI308-11, TRIFI934-13 |
|  | Germany | KX291065, KX295073 | GBEPT2152,-15 GBEPT816-14 |
|  | Iceland | HM395687 | KKCAD577-09 |
|  | Norway | COI-5P, HM395674, HM395675, HM395676, KX295389 | KKCAD551-09, KKCAD552-09, KKCAD553-09, KKCAD555-09, TRDTR052-14 |
| *Limnephilus elegans* | Czechia | KX295211 | BHMKK249-12 |
|  | Finland | HM902366, HM902367, KX142371 | FICAD036-10, FICAD037-10, TRIFI306-11 |
|  | Iceland | NA | ICEFI003-17 |
| *Limnephilus griseus* | Croatia | NA | CROAA125-18 |
|  | Czechia | HQ958957, HQ958979, KX291336 | BHMKK173-12, HMKKT058-10, HMKKT082-10 |
|  | Finland | HM902368, KX142109, KX292184, KX294074 | FICAD039-10, TRIFI272-10, TRIFI829-12 ,TRIFI946-13 |
|  | Germany | KX291390, KX292189, KX295660, KX296358 | FBAQU1197-12, FBAQU1198-12 ,GBEPT771-14, GBEPT772-14 |
|  | Iceland | HM395681, HM395682, HM395689, HM395690, HM395693 | KKCAD568-09, KKCAD569-09, KKCAD584-09, KKCAD585-09, KKCAD590-09 |
|  | Norway | KX105440, KX292103 | FINNT037-12, NWCAD004-08 |
|  | Russia | NA | TRIFI1211-15 |
|  | Scotland | KX105440 NA | NMS371-23, NWCAD004-08 |
|  | Switzerland | NA | PLEAA954-20, PLEAA955-20 |
| *Limnephilus sparsus* | Austria | HQ959128, HQ959320, HQ959329, HQ959330, KX104132, KX104550 ,KX143714 | HMCAD495-09, HMKKT247-10, HMKKT258-10, HMKKT546-10, HMKKT557-10, HMKKT558-10, HMTRI273-09, INTAP118-17, INTAP119-17, INTAP131-17 |
|  | Belgium | KX143929, KX144559, KX144605 KX144833 | TFLAN191-11, TFLAN192-11, TFLAN193-11, TFLAN277-11 |
|  | Croatia | NA | CROTR001-19, CROTR025-19 |
|  | Czech-Republic | KX295922 | BHMKK167-12 |
|  | England | NA | UKAN2920-24 |
|  | Finland | HM902374, KX295210, KX295837 | FICAD050-10, TRIFI1181-15, TRIFI920-13 |
|  | Germany | KX293584, KX295793, KX296438 | FBAQU1203-12, GBEPT2170-15, GBEPT774-14 |
|  | Iceland | HM395684, HM395685, HM395686 | KKCAD572-09, KKCAD573-09, KKCAD574-09 |
|  | Japan | KX102901, KX105167, KX106912 | JPCAD349-08, JPCAD350-08, JPCAD351-08 |
|  | Macedonia | NA | JHTRI056-19, JHTRI057-19 |
|  | Mongolia | KX103331, KX104078, KX104095, KX104838, KX105623, KX105692, KX106571, KX107207 | MGCAD346-08, MGCAD347-08, MGCAD348-08, MGCAD349-08, MGCAD350-08, MGCAD351-08, MGCAD352-08, MGCAD353-08 |
|  | Norway | KX106170, KX106713, KX106914, KX293408, KX293800, KX294405, KX294574 ,KX295957 | JPCAD347-08, NWCAD002-08, NWCAD005-08, ODTRI066-14, ODTRI067-14, ODTRI068-14, TRDTR163-15, TRDTR164-15 |
|  | Portugal | OP817672, OP817683, OP817701, OP817843 | IBITR079-20, IBITR153-20, IBITR154-20, IBITR171-20 |
|  | Scotland | NA | NMS364-23, UKAN4919-24 |
|  | Spain | OP817947 | IBITR255-20 |
|  | Switzerland | NA | IBITR255-20 |
|  | Austria | KX291422 | KJTRI120-13 |
|  | Czech Republic | AB971912, HQ958958, HQ958978, KX291336, KX294468 | BHMKK171-12, BHMKK173-12, GBMNA10390-19, HMKKT060-10, HMKKT081-10 |
|  | Estonia | KX296228 | TRIFI1139-15 |
|  | Finland | COI-5P, HM902354, MZ624808, KX144316 | COLFH1615-16, FICAD018-10, FICAD022-10, TRIFI294-11 |
|  | Iceland | COI-5P, HM395678, HM395694 | ICEFI002-17, KKCAD565-09, KKCAD591-09 |
|  | Switzerland | NA | PLEAA938-20, PLEAA939-20 |
| *Micropterna sequax* | Austria | HQ959114.1 | NA |
|  | Belgium | KX140874.1 | NA |
|  | Denmark | MT483685.1 | NA |
|  | Finland | KX141807.1, KX292376.1, KX296118.1, KX296119.1, MZ626070.1, MZ627905.1 | NA |
|  | Iceland | ICEFI001-17 | NA |
|  | Norway | KX292191.1, KX293161.1, KX295801.1, KX296216.1 | NA |
|  | San Marino | KX142211.1 | NA |
|  | Spain | MW579506.1 | NA |
|  | UK | KY225462.1, KY225467.1 | NA |
